# Supplementary material for: Bayesian Hierarchical Modelling of Historical Data of the South African Coal Mining Industry for Compliance Testing
Source: Int J Environ Res Public Health. 2022 Apr 7;19(8):4442. doi: 10.3390/ijerph19084442 (PMC9032634; doi:10.3390/ijerph19084442)
Supplement: Supplementary file 1 [file ijerph-19-04442-s001.zip › ijerph-1584586-supplementary.pdf]

Supplement for: A Method for Constructing Informative Priors for Bayesian Modeling of Occupational Hygiene Data

Harrison Quick[*1], **Tran Huynh**[2], **and Gurumurthy Ramachandran**[3]

[1] Department of Epidemiology and Biostatistics, Drexel University, Philadelphia, PA 19104

[2] Department of Environmental and Occupational Health, Drexel University, Philadelphia, PA 19104

[3] Department of Environmental Health Sciences, Johns Hopkins University, Baltimore, MD 21205

[*] *email:* hsq23@drexel.edu

This is the supplement for "A method for constructing informative priors for Bayesian modeling of occupational hygiene data" by Harrison Quick, Tran Huynh, and Gurumurthy Ramachandran. Section 1 offers a detailed description of the prior distributions presented in the main manuscript. Section 2 discusses the motivations for and implications of bounds on GM and GSD compared to bounds on $X_{0.95}$. In Section 3, we conduct a simulation study to evaluate the performance of the informative priors we discuss. Section 4 provides R code which can be used to replicate the illustrative examples from the manuscript.

1. Prior Distributions

In this section, we offer a brief discussion on the subject of noninformative priors and present a more detailed derivation of the informative priors given in (3) and (4). We will also demonstrate how these priors could arise as the posterior distribution based on historical data, y_{i0}, $i = 1, \ldots, n_0$, and noninformative priors as an additional motivation for choosing these specific prior distributions. Finally, this section concludes with derivations of the full conditional distributions for μ and σ^2, which are then used in the MCMC algorithm found in Section 4.

1.1. Noninformative Priors

Before diving too deeply into the origins of the *informative* priors presented in the paper, it is important to more clearly articulate what it means to be a *noninformative* prior. From the expression for Bayes' Theorem in (1) of the main manuscript, we know

$$p(\boldsymbol{\theta} \,|\, \mathbf{Y}) \propto p(\mathbf{Y} \,|\, \boldsymbol{\theta}) \times p(\boldsymbol{\theta}). \qquad \text{(S1)}$$

Note the use of blue-shaded text to denote information contributed by the data and orange-shaded text to denote information contributed by the prior. Using the example from the paper, let's assume $\mathbf{Y} = (Y_1, \ldots, Y_n)'$ and $Y_i \sim \text{Norm}(\mu, \sigma^2)$, where μ denotes the log of the geometric mean (GM) and σ denotes the log of the geometric standard deviation (GSD). For the sake of illustration, suppose σ is known and $\boldsymbol{\theta} = \mu$ is the only unknown parameter in our

model. Updating (S1), we find that the posterior distribution for μ is

$$p(\mu \mid \mathbf{Y}) \propto \prod_{i=1}^{n} \frac{1}{\sqrt{2\pi\sigma^2}} \exp\left(-\frac{(Y_i-\mu)^2}{2\sigma^2}\right) \times p(\mu), \tag{S2}$$

where $p(\mu)$ denotes the prior distribution of μ. Finally, note that since μ is the mean of a normal distribution, thus any value $\mu \in (-\infty, \infty)$ is *theoretically* valid. We will now explore a few of the claims from the Background section of the paper.

1.1.1. Uniform Priors for μ

Suppose, as proposed in (2), we assume μ is uniformly distributed over the range $\mu \in (a_\mu, b_\mu)$; i.e.,

$$\begin{aligned} p(\mu) &= \frac{1}{b_\mu - a_\mu} \times I\{\mu \in (a_\mu, b_\mu)\} \\ &\propto I\{\mu \in (a_\mu, b_\mu)\}. \end{aligned} \tag{S3}$$

This leads to a posterior distribution of the form

$$p(\mu \mid \mathbf{Y}) \propto \prod_{i=1}^{n} \frac{1}{\sqrt{2\pi\sigma^2}} \exp\left(-\frac{(Y_i-\mu)^2}{2\sigma^2}\right) \times I\{\mu \in (a_\mu, b_\mu)\}. \tag{S4}$$

In particular, note that the contribution of the prior for μ is limited to the constraint that $\mu \in (a_\mu, b_\mu)$. That is, if the range (a_μ, b_μ) is sufficiently extreme, the posterior distribution for μ will (in essence) contain *no* information from the prior distribution. On the other hand, now suppose $\mu = \ln \mathrm{GM} \in (a_\mu, b_\mu)$ corresponds to an interval of low exposure levels, while our sample of data suggests more intermediate exposure levels for μ. With this prior restriction, however, the posterior distribution in (S4) will reside entirely in the low exposure range. Thus, despite the use of a uniform prior distribution for μ, the use of restrictive bounds can lead to $\mu \sim \mathrm{Unif}(a_\mu, b_\mu)$ being a quite *informative* prior distribution.

1.1.2. Uniform Priors for $\mathrm{GM} = e^\mu$

Now suppose we assume $\mathrm{GM} = e^\mu$ is uniformly distributed over the range $\mu \in (a_\mu, b_\mu)$; i.e., we use the same set of bounds, but instead of (S3), we assume

$$\begin{aligned} p_{\mathrm{GM}}(\mathrm{GM}) &= \frac{1}{e^{b_\mu} - e^{a_\mu}} \times I\left\{\mathrm{GM} \in \left(e^{a_\mu}, e^{b_\mu}\right)\right\} \\ &\propto I\left\{\mathrm{GM} \in \left(e^{a_\mu}, e^{b_\mu}\right)\right\}. \end{aligned} \tag{S5}$$

Since the likelihood in (S2) is a function of μ, however, it may be helpful to use a variable transformation to obtain the prior distribution for μ. Note that this requires deriving the expression for the *Jacobian* of the transformation $\mathrm{GM} = e^\mu$, which is equal to

$$J(\mu) = \left|\frac{d\mathrm{GM}}{d\mu}\right| = e^\mu.$$

From this, we find that the prior distribution, as a function of μ, is of the form:

$$\begin{aligned} p(\mu) &= p_{\text{GM}}\left(\text{GM} = e^{\mu}\right) \times J(\mu) \\ &= \frac{1}{e^{b_\mu} - e^{a_\mu}} \times I\left\{e^{\mu} \in \left(e^{a_\mu}, e^{b_\mu}\right)\right\} \times e^{\mu} \\ &\propto e^{\mu} \times I\left\{\mu \in (a_\mu, b_\mu)\right\}. \end{aligned} \tag{S6}$$

This leads to a posterior distribution for μ of the form:

$$p(\mu \mid \mathbf{Y}) \propto \prod_{i=1}^{n} \frac{1}{\sqrt{2\pi\sigma^2}} \exp\left(-\frac{(Y_i - \mu)^2}{2\sigma^2}\right) \times e^{\mu} \times I\left\{\mu \in (a_\mu, b_\mu)\right\}. \tag{S7}$$

As you can see, the contribution of the prior in the posterior distribution in (S7) is more than the simple constraint on the bounds, thus GM $\sim$ Unif$\left(e^{a_\mu}, e^{b_\mu}\right)$ could bias our results and thus should *not* be considered a noninformative prior.

1.1.3. Noninformative Priors for σ^2

Unlike the mean parameter, μ, in a normal likelihood, there is not a single standard noninformative prior for the parameter σ^2. Rather than discuss this subject in detail, however, we will consider uniform priors on σ with relaxed bounds to be noninformative and refer more curious practitioners to the work of Gelman (2006). In particular, Gelman (2006) favors a uniform prior on σ (which yields $p(\sigma^2) \propto \sigma^{-1}$) over the seemingly less informative uniform prior on σ^2 (which yields $p(\sigma^2) \propto 1$).

1.2. An Informative Prior for μ

1.2.1. Based on Frequentist Properties

In the classical frequentist setting, it can be shown that $\bar{y} \sim \text{Norm}(\mu, \sigma^2/n_0)$, where μ and σ^2 are considered fixed, unknown parameters. This can then be restructured into

$$\frac{\bar{y}_0 - \mu}{\sigma/\sqrt{n_0}} \sim \text{Norm}(0, 1).$$

Given that we know $\bar{y}_0$, s^2_{y0}, and n_0 from our historical data, we can replace σ^2 with its estimate, s^2_{y0}, and obtain

$$\mu \sim \text{Norm}(\bar{y}_0, s^2_{y0}/n_0). \tag{S8}$$

1.2.2. Comparison to Historical Data Posterior

A common noninformative prior for μ is

$$p(\mu) \propto 1,$$

that is, a *flat* prior. Using this prior will then lead to

$$p(\mu \mid \cdot) \propto \prod_{i=1}^{n_0} N(y_{i0} \mid \mu, \sigma^2)$$
$$\propto \exp\left[-\frac{\sum(y_{i0}-\mu)^2}{2\sigma^2}\right]$$
$$\propto \exp\left[-\frac{(\bar{y}_0-\mu)^2}{2\sigma^2/n_0}\right]$$

which yields a full-conditional distribution of

$$\mu \mid \sigma^2, \mathbf{Y}_0 \sim N(\bar{y}_0, \sigma^2/n_0).$$

Replacing σ^2 with its estimator, s^2_{y0}, as before, we find that this distribution is equivalent to the intuitive prior in (S8).

1.3. *An Informative Prior for σ^2*

1.3.1. Based on Frequentist Properties

To construct our informative prior for σ^2, we begin with the expression

$$(n_0-1)s^2_{y0}/\sigma^2 \sim \chi^2_{n_0-1}.$$

Note that $\chi^2_{n_0-1} \equiv \text{Gam}(\frac{n_0-1}{2}, 2)$, where the second parameter in the gamma distribution is the scale parameter. Then, using the properties of the gamma distribution, we find

$$(n_0-1)s^2_{y0}/\sigma^2 \sim \text{Gam}\left(\frac{n_0-1}{2}, 2\right)$$
$$\implies \frac{1}{\sigma^2} \sim \text{Gam}\left(\frac{n_0-1}{2}, \frac{2}{(n_0-1)s^2_{y0}}\right)$$
$$\implies \sigma^2 \sim \text{IG}\left(\frac{n_0-1}{2}, \frac{(n_0-1)s^2_y}{2}\right), \tag{S9}$$

where if $X \sim \text{Gam}(a, b)$, then $1/X \sim \text{IG}(a, 1/b)$.

1.3.2. Comparison to Historical Data Posterior

Following Gelman (2006), we choose to use the noninformative prior $\sigma \sim \text{Unif}(0, A)$, where we assume $A \to \infty$ (or that A is some arbitrarily large number). Transforming this prior to the σ^2 scale, we obtain $p(\sigma^2) \propto 1/\sigma$. Using this prior, we find

$$p(\sigma^2 \mid \cdot) \propto \prod_{i=1}^{n_0} \text{Norm}(y_{i0} \mid \mu, \sigma^2) 1/\sigma$$
$$\propto (\sigma^2)^{-n_0/2} \exp\left[-\frac{\sum(y_{i0}-\mu)^2}{2\sigma^2}\right] (\sigma^2)^{-1/2}$$
$$\propto (\sigma^2)^{-(n_0-1)/2-1} \exp\left[-\frac{\sum(y_{i0}-\mu)^2}{2\sigma^2}\right],$$

which gives the full-conditional distribution of

$$\sigma^2 \mid \mu, \mathbf{Y}_0 \sim \text{IG}\left(\frac{n_0 - 1}{2}, \frac{\sum(y_{i0} - \mu)^2}{2}\right).$$

Replacing μ with its estimator, $\bar{y}_0$, as before and noting that $(n-1)s_{y0}^2 = \sum(y_{i0} - \bar{y}_0)^2$, we find that this distribution is equivalent to the prior in (S9).

1.4. Full-Conditional Distributions

We now show the derivations for the full-conditional distributions for μ and σ^2 based on the truncated normal and truncated inverse gamma prior distributions. These will be used in the R code in Section 4.

1.4.1. Full-Conditional for μ

$$\begin{aligned} p\left(\mu \mid \mathbf{Y}, \sigma^2, \bar{y}_0, s_{y0}^2\right) &\propto p\left(\mathbf{Y} \mid \mu, \sigma^2\right) \times p\left(\mu \mid \bar{y}_0, s_{y0}^2\right) \\ &\propto \exp\left[-\frac{(\bar{y} - \mu)^2}{2\sigma^2/n}\right] \times \exp\left[-\frac{(\bar{y}_0 - \mu)^2}{2s_0^2/n_0}\right] \times I\left\{\mu \in [a_\mu, b_\mu]\right\} \\ &\propto \exp\left[-\frac{(\mu - E[\mu \mid \cdot])^2}{2V[\mu \mid \cdot]}\right] \times I\left\{\mu \in [a_\mu, b_\mu]\right\} \end{aligned}$$

where $E[\mu \mid \cdot] = V[\mu \mid \cdot]\left(n\bar{y}/\sigma^2 + n_0\bar{y}_0/s_{y0}^2\right)$ and $V[\mu \mid \cdot] = \left(n/\sigma^2 + n_0/s_{y0}^2\right)^{-1}$. This indicates that

$$\mu \mid \mathbf{Y}, \sigma^2, \bar{y}_0, s_{y0}^2 \sim \text{Trun-Norm}\left(E[\mu \mid \cdot], V[\mu \mid \cdot]\right)[a_\mu, b_\mu]. \qquad \text{(S10)}$$

1.4.2. Full-Conditional for σ^2

$$\begin{aligned} p\left(\sigma^2 \mid \mathbf{Y}, \mu, \bar{y}_0, s_{y0}^2\right) &\propto p\left(\mathbf{Y} \mid \mu, \sigma^2\right) \times p\left(\sigma^2 \mid \bar{y}_0, s_{y0}^2\right) \\ &\propto \left(\sigma^2\right)^{-\frac{n+n_0-1}{2}-1} \exp\left[-\frac{n(\bar{y} - \mu)^2 + (n_0 - 1)s_{y0}^2}{2\sigma^2}\right] \times I\left\{\sigma^2 \in [a_\sigma, b_\sigma]\right\}. \end{aligned}$$

This indicates that

$$\sigma^2 \mid \mathbf{Y}, \mu, \bar{y}_0, s_{y0}^2 \sim \text{Trun-IG}\left(\frac{n + n_0 - 1}{2}, \frac{n(\bar{y} - \mu)^2 + (n_0 - 1)s_{y0}^2}{2}\right)[a_\sigma, b_\sigma] \qquad \text{(S11)}$$

2. Comparison of Bounds

This section consists of a comparison of bounds based on GM and GSD and bounds based on $X_{0.95}$. In our paper, we argue our belief that bounds on the parameters should be placed based on *decision-making* criteria, rather than as a means of incorporating prior information into the model. Here, we hope to expand on that idea. Before we make a *quantitative* comparison, however, we look to compare and contrast the potential motivation for each specification of bounds.

2.1. Motivation for Bounds

2.1.1. Bounds based on GM and GSD

When directly specifying bounds on GM and GSD in an occupational hygiene setting, whether using the prior distributions proposed by Hewett et al. (2006), those used by Huynh et al. (2016), or otherwise, a common theme can be observed. The bounds proposed for GM tend to be quite broad, covering a wide range of possible values. This is important because if knowledge is lacking about the true value of GM, then we need a prior which is flexible enough to accommodate this. Fortunately, $\mu = \log \text{GM}$ is typically well estimated from a model, as evidenced by how common the parameter is given a noninformative prior in nearly all Bayesian models — e.g., many of the examples in software packages like OpenBUGS (Lunn et al., 2009). That is (loosely speaking), prior precision is not necessarily *required* to obtain relatively precise posterior estimates of GM (e.g., note that the upper bounds for GM in Table 2 do not differ substantially, despite the differences in prior information).

Thus, it is the upper bound for GSD that is of the utmost importance. Here, the bound is often based on "rule-of-thumb" criteria, such as the suggestion that GSD > 4 corresponds to a "poorly defined" exposure group (EG; e.g., see Chapter 4 of Mulhausen et al., 2006; Chapter 16 of Ramachandran, 2005). The implication here is that it's unclear how to proceed if the data suggests GSD ≈ 4 or GSD > 4 — i.e., if it is not feasible to collect more data to better estimate GSD, do we increase this bound (thereby dropping the assumption of a well-defined EG), or simply classify these EGs as poorly defined?

2.1.2. Bounds based on $X_{0.95}$

The prior distributions proposed in this paper are inspired by the setting where our decision-making criteria is to estimate the most appropriate AIHA exposure category, categories which are based on the ratio of the 95th percentile, $X_{0.95} = \exp[\mu + 1.645\sigma]$, and the predefined occupational exposure limit (OEL). In this scheme, the highest exposure category is defined as $X_{0.95} > \text{OEL}$, with lower values of $X_{0.95}$ corresponding to lower exposure categories. Here, we opted to bound $X_{0.95} < 2 \times \text{OEL}$, operating under the assumption that (a) if $X_{0.95} \ll \text{OEL}$, we will be able to identify the correct exposure category and (b) if $X_{0.95}$ is close to or greater than the OEL, we can increase our bound on $X_{0.95}$ to assess our posterior distribution's sensitivity to the bound. That is, the bound on $X_{0.95}$ is purely intended to improve the precision of our estimates via restricting the parameter space, not a reflection of our prior *beliefs* (in the conventional sense of the word; e.g., see Gelman, 2015). Thus, if the posterior distribution is overly constrained by the $X_{0.95} < 2 \times \text{OEL}$ restriction, we can relax this bound without considering the implications of relaxing the bound on GSD.

2.2. Illustrative Example, Revisited

Given these potential motivations, we revisit the illustrative example from the main paper, using Past Sample A to provide estimates of GM and GSD. The top row of Figure S1 displays scatterplots of the samples from the prior and posterior distributions of our truncated normal and truncated inverse gamma distributions where bounds have been imposed directly on GM and GSD compared to bounds on $X_{0.95}$. Here, transparent circles denote samples

Table S1. Posterior estimates from the various prior specifications.

Prior	Median (95% CI) GM = 1	GSD = 2.71	$X_{0.95} = 5.18$	AIHA Category Probabilities # 2	# 3	# 4
Standard						
GM/GSD	0.91 (0.46, 1.97)	2.23 (1.60, 3.73)	3.42 (1.38, 12.81)	0.52	0.34	0.14
$X_{0.95}$	0.90 (0.45, 1.80)	2.25 (1.60, 4.32)	3.49 (1.39, 11.91)	0.51	0.33	0.15
Relaxed						
GM/GSD	0.93 (0.46, 2.13)	2.31 (1.61, 6.12)	3.73 (1.40, 26.37)	0.47	0.31	0.22
$X_{0.95}$	0.92 (0.46, 2.02)	2.30 (1.61, 5.48)	3.67 (1.40, 21.12)	0.48	0.31	0.21

from the prior distribution, opaque circles denote samples from the posterior distribution, and the shades of red denote the various AIHA exposure categories. Despite the data being generated from a distribution with GM = 1, GSD = 2.71, and $X_{0.95} = 5.18$ (AIHA Category 3), both posterior distributions are clearly affected by the restricted parameter spaces. The top two rows of Table S1 display the posterior probabilities associated with each of these prior specifications.

We now consider relaxing the prior restrictions to GSD < 10 and $X_{0.95} < 5 \times$ OEL. The bottom row of Figure S1 displays the new scatterplots, and the bottom two rows of Table S1 display the new posterior probabilities. First and foremost, it appears as though the informative prior structure (i.e., the normal and inverse gamma distributions) ensures that the results remain comparable, irrespective of the bounds imposed. That is, while both approaches are effective for restricting the parameter space and obtaining more precise estimates than with an unbounded prior, placing the restriction on $X_{0.95}$ allows us to evaluate the posterior probability that GSD exceeds 4 — i.e., $P\left(\text{GSD} > 4 \mid \mathbf{Y}\right)$ — without the need to change our prior assumption that the data is from a well-formed exposure group. It should be noted that this similarity should be expected provided the bounds are sufficiently far from the posterior medians, though this may not be the case when (say) the data suggest larger values of GSD are required. Secondly, note that the distributions in Figures S1c,d are *still* being restricted, despite using the more relaxed bounds.

While not shown here, the results obtained using bounded *uniform* priors would be substantially different, with increased variability for both GM and GSD, as would be inferred by Tables 2 and 3 in the main manuscript and Tables S2–S4 in this supplement. That said, the goal of this example was to illustrate the effect of the *bounds*, not the *informativeness* of the prior distributions.

3. Simulation Study

In order to reinforce the findings of the illustrative example, we have designed a simulation study which compares the performance of these priors to the bounded uniform priors conventionally used in the literature. More specifically, we again wish to analyze a dataset comprised of $n = 3$ observations generated from a lognormal distribution with GM = 1 and GSD = 2.71;

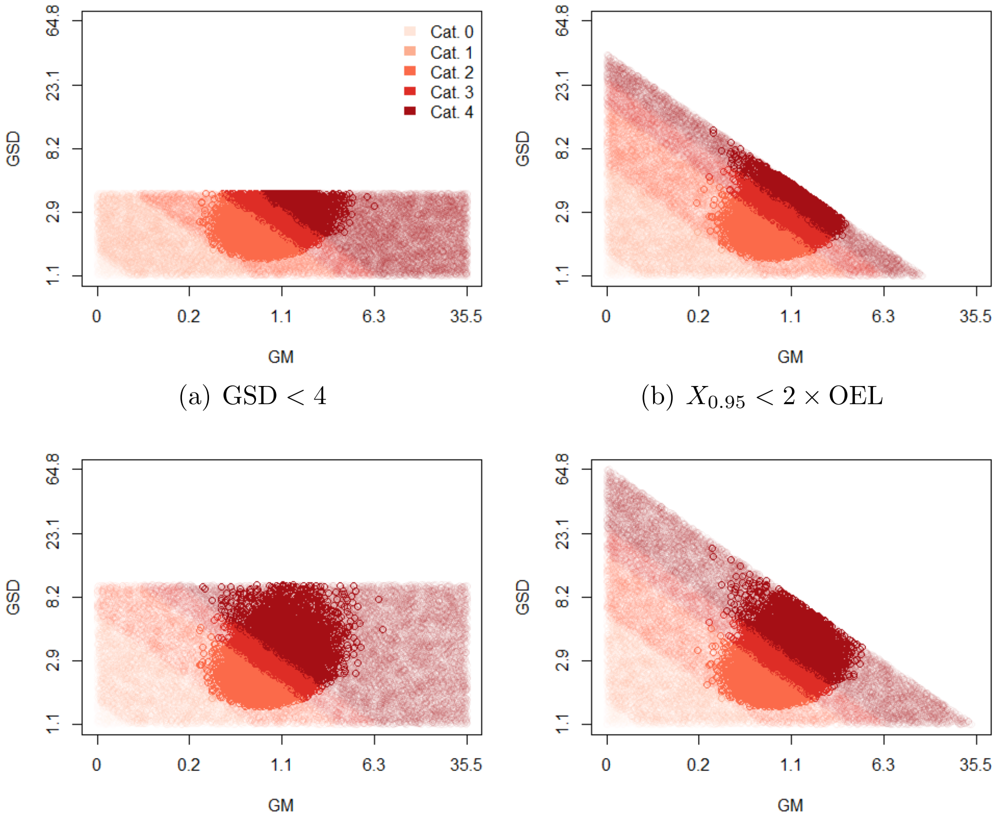

(a) GSD < 4

(b) $X_{0.95} < 2 \times$ OEL

(c) GSD < 10

(d) $X_{0.95} < 5 \times$ OEL

Figure S1. Comparison of posterior distributions based on bounds placed on GM and GSD and bounds placed on $X_{0.95}$ for two sets of bounds. Distributions in the top row correspond to the bounds featured in the illustrative example in the main manuscript; i.e., GM $\in$ and GSD $\in$ (1.05, 4) and $X_{0.95} < 2 \times$ OEL. Distributions in the bottom row correspond to more relaxed prior bounds, specifically GSD $\in$ (1.05, 10) and $X_{0.95} < 5 \times$ OEL. Transparent circles denote the range of samples from the prior distribution, while opaque circles denote the samples from the posterior distribution.

Table S2. Root mean square error for the parameters of interest — GM, GSD, and X0.95, as well as their natural logs — for each of our prior specifications.

	Data set	GM = 1	GSD = 2.71	$X_{0.95} = 5.18$
	A	0.48	1.21	3.40
$X_{0.95} \leq 2 \times \text{OEL}$	B	0.57	0.87	4.05
	C	0.51	1.98	3.13
Uniform ($X_{0.95}$)	—	0.60	2.65	3.47
Uniform (GM, GSD)	—	0.84	0.69	6.28
		$\mu = 0$	$\sigma = 1$	$\log X_{0.95} = 1.645$
	A	0.53	0.32	0.56
$X_{0.95} \leq 2 \times \text{OEL}$	B	0.48	0.27	0.57
	C	0.79	0.45	0.58
Uniform ($X_{0.95}$)	—	0.80	0.52	0.64
Uniform (GM, GSD)	—	0.66	0.27	0.80

i.e., logNorm(0, 1). To construct our informative prior distributions, we use estimates of GM and GSD based on past data generated from one of *three* distributions — logNorm(0, 1) (denoted dataset "A"), logNorm(1, 1) ("B"), or logNorm(−1, 1) ("C"). Given these estimates, we will construct the truncated normal and truncated inverse gamma priors described in equations (5) and (6), constrained such that $X_{0.95}$ is less than two times the OEL. In addition to these prior distributions, we will consider two sets of uniform priors: one where bounds for GM and GSD are based on $X_{0.95}$ (i.e., the noninformative version of our informative priors) and one where GM $\in$ (OEL/200, 5 $\times$ OEL) and GSD $\in$ (1.05, 4). Based on the AIHA expo-sure categories given in Table C.2 and given that the true $X_{0.95} = \exp\left[0 + 1.645 \times 1\right] = 5.18$, we investigate two potentially concerning scenarios: OEL $= \exp\left[0 + 1.96 * 1\right] = 7.09$ (i.e., AIHA Category 3) and OEL $= \exp\left[0 + 1.24 \times 1\right] = 3.45$ (i.e., AIHA Category 4). Each combination of prior specification and exposure scenario is investigated using 100 simulated data sets.

Table S2 presents the root mean square error (rMSE) for GM, GSD, and $X_{0.95}$ using our various prior specifications. More specifically, we compute

$$\text{rMSE}\left(\theta\right) = \sqrt{\frac{\sum_{\ell=1}^{100}\left(\theta^{(\ell)} - \theta\right)^2}{100}},$$

where $\theta^{(\ell)}$ denotes the posterior median of the parameter, θ, from the ℓ-th simulated data set. Due to the bounded and skewed nature of many of these parameters (e.g., all non-zero with long right tails), we also computed the rMSE for the log-transformed parameters — this will help alleviate the disproportionate penalization of overestimation. From these results, it appears the informative priors proposed here consistently outperform the uniform priors, both on the scale of interest and the log-transformed scale. The primary exception to this is the uniform prior specification in which GSD is restricted to be less than 4, where this upper bound forces the posterior distribution to remain close to the true value.

Next, we turn our attention to the decision making aspect of the illustrative example in

the main manuscript — here again, we assume the OEL = 7.09, resulting in a Category 3 AIHA exposure scenario. In Table S3, which displays the median (95% CI) of the posterior probability for each exposure category, we find that the priors that yield the highest probability in Category 3 are our three sets of informative priors, each of which has a greater than 36% chance of identifying the correct exposure category. With the exception of when our past data underestimate the GM (i.e., past data set C), more than 70% of our posterior mass for these informative priors falls within Categories 3 and 4, indicating that these priors can be considered to be rather conservative from a regulatory perspective, while the uniform prior specifications tend to yield results that are more favorable toward lower exposure categories.

Finally, Table S4 displays the results for the Category 4 scenario. As in the Category 3 scenario, the uniform prior specifications tend to yield posterior distributions which favor smaller values for $X_{0.95}$, resulting in lower Category 4 probabilities. Perhaps more troubling, however, is the variability observed in these probabilities — while all of our simulated data sets resulted in a majority of the posterior mass in either Categories 3 or 4 for the informative prior specifications, the uniform prior specifications occasionally led to nearly 70% of the posterior mass in Category 2 and just 10% of the posterior mass in Category 4. In other words, there can be substantial variability in decision making when based on only $n = 3$ observations, but this issue can be mitigated by the infusion of prior information.

4. R Code

The following code can be used to recreate the results from the informative priors in the illustrative example.

```
#remove all existing variables from R's memory
rm(list=ls())
#set a random number seed in order to achieve reproducibility
set.seed(630)
#set the sample size of the current data (N),
#the past data (N0),
#and the prior sample size (n0)
N=3; N0=5; n0=3

########################
#Past Data
########################
mu0=0; sig0=1  #Past Data A
#mu0=1; sig0=1 #Past Data B
y0=rnorm(N0,mu0,sig0)
ybar0=mean(y0)
sy20=var(y0)

########################
```

	Data set	Category 2 $\frac{\text{OEL}}{10} < X_{0.95} \leq \frac{\text{OEL}}{2}$	**Category 3** $\frac{\text{OEL}}{2} < X_{0.95} \leq \text{OEL}$	Category 4 $X_{0.95} > \text{OEL}$
	#1	0.176 (0.015, 0.518)	**0.397 (0.246, 0.498)**	0.384 (0.153, 0.736)
$X_{0.95} \leq 2 \times \text{OEL}$	#2	0.080 (0.003, 0.352)	**0.366 (0.153, 0.451)**	0.540 (0.277, 0.841)
	#3	0.312 (0.054, 0.705)	**0.396 (0.208, 0.485)**	0.257 (0.078, 0.601)
Uniform ($X_{0.95}$)	—	0.351 (0.030, 0.801)	**0.332 (0.074, 0.464)**	0.274 (0.061, 0.718)
Uniform (GM, GSD)	—	0.381 (0.012, 0.844)	**0.314 (0.064, 0.425)**	0.257 (0.026, 0.854)

Table S3. Estimated exposure category probabilities when the true scenario is Category 3. Category 1, where $X_{0.95} \leq \frac{\text{OEL}}{10}$, has been removed for space concerns.

	Data set	Category 2<br>$\frac{OEL}{10} < X_{0.95} \leq \frac{OEL}{2}$	Category 3<br>$\frac{OEL}{2} < X_{0.95} \leq OEL$	**Category 4**<br>$X_{0.95} > \textbf{OEL}$
	#1	0.014 (0.000, 0.184)	0.260 (0.061, 0.465)	**0.730 (0.384, 0.939)**
$X_{0.95} \leq 2 \times OEL$	#2	0.005 (0.000, 0.079)	0.164 (0.020, 0.390)	**0.833 (0.515, 0.980)**
	#3	0.043 (0.002, 0.332)	0.366 (0.130, 0.503)	**0.599 (0.234, 0.867)**
Uniform ($X_{0.95}$)	—	0.062 (0.000, 0.670)	0.344 (0.078, 0.578)	**0.513 (0.096, 0.917)**
Uniform (GM, GSD)	—	0.046 (0.000, 0.689)	0.282 (0.009, 0.492)	**0.633 (0.096, 0.991)**

Table S4. Estimated exposure category probabilities when the true scenario is Category 4. Category 1, where $X_{0.95} \leq \frac{OEL}{10}$, has been removed for space concerns.

```
#New Data
########################
mu1=0; sig1=1
x=rnorm(N,mu1,sig1)

oel=exp(mu1+1.96*sig1)
thres=2 #upper bound for X95

########################
#Prior Specifications
########################
#n0=N;
m0=ybar0; v0=sy20

#mu ~ N(theta,tau2)
theta=m0; tau2=v0/n0
#theta=0; tau2=Inf #This yields a noninformative prior
#bounds for mu
#While a lower bound of -Inf would be perfectly valid,
#we use the lower bound suggested by Hewett et al. (2006).
#Fortunately, this value is sufficiently small so as to
#have ~no impact on our results, whatsoever.
#As for the upper bound, that will change iteratively below
mub=c(log(.005*oel),NA)

##################
#sig2 ~ IG(a,b)
#Note that R uses an alternate parameterization of the IG
a=(n0-1)/2; b=2/(v0*(n0-1)) #second parameter is inverted
#a=-1/2; b=Inf #This corresponds to a flat prior on sig=log(GSD)
#              #(see Gelman 2006)
#As with mu, we use the lower bound from BDA
#and let the upper bound vary iteratively
sigb=c(log(1.05),NA)^2

#####################
#The MCMC algorithm
#####################

niter=20000 #20,000 iterations
mu=sig=sig2=rep(NA,niter)
#initialize our parameters
mu[1]=0
sig[1]=sig2[1]=1
```

```
for(i in 2:niter){
#specify upper bound for mu based on most recent value of sigma
  mub[2]=log(thres*oel)-1.645*sig[i-1]
#update mu
  mumu = (mean(x)/(sig2[i-1]/N) + theta/tau2) / (N/sig2[i-1] + 1/tau2)
  musig= 1/(N/sig2[i-1] + 1/tau2)
#since we're using a truncated normal,
#we use the inverse CDF method to sample mu
#if mub[1] < mu < mub[2],
#then pnorm(mub[1]) < pnorm(mu) < pnorm(mub[2])
#where pnorm(.) denotes the appropriate CDF
  u=runif(1,pnorm(mub[1],mumu,sqrt(musig)),
            pnorm(mub[2],mumu,sqrt(musig)))
  mu[i]=qnorm(u,mumu,sqrt(musig))

#specify upper bound for sigma^2 based on new value of mu
  sigb[2]=( (log(thres*oel)-mu[i])/1.645 )^2
#update sig2
#again using the inverse CDF method
  u=runif(1,pgamma(1/sigb[2],N/2+a, (1/b+sum( (x-mu[i])^2 )/2)),
            pgamma(1/sigb[1],N/2+a, (1/b+sum( (x-mu[i])^2 )/2)))
  sig2[i]=1/qgamma(u,N/2+a, (1/b+sum( (x-mu[i])^2 )/2) )
  sig[i]=sqrt(sig2[i])
}

#compute x95 using the samples of mu, sig
x95=mu+1.645*sig

#####################
#Compute exposure category probabilities
#####################

#compute boundaries of the exposure categories
A=c(log(0.01*oel),log(0.10*oel),log(0.50*oel),log(oel))

catl=array(rep(x95,times=4)>rep(A,each=niter),dim=c(niter,4))
cat.post=apply(catl,1,sum)
table(cat.post)/niter
#cat.post
#      1       2       3       4
#0.00010 0.51185 0.33440 0.15365
```

References

Gelman, A. (2006). "Prior distributions for variance parameters in hierarchical models." *Bayesian Analysis*, 1, 515–533.

— (2015). "Prior *information*, not prior *belief*." `http://andrewgelman.com/2015/07/15/prior-information-not-prior-belief/`. Accessed Feb. 21, 2016.

Hewett, P., Logan, P., Mulhausen, J., Ramachandran, G., and Banerjee, S. (2006). "Rating exposure control using Bayesian Decision Analysis." *Journal of Occupational and Environmental Hygiene*, 3, 568–581.

Huynh, T., Quick, H., Ramachandran, G., Banerjee, S., Stenzel, M., Sandler, D. P., Engel, L. S., Kwok, R. K., Blair, A., and Stewart, P. A. (2016). "A comparison of the β-substitution method and a Bayesian method for analyzing left-censored data." *Annals of Occupational Hygiene*, 60, 56–73.

Lunn, D., Spiegelhalter, D., Thomas, A., and Best, N. (2009). "The BUGS project: Evolution, critique, and future directions." *Statistics in Medicine*, 28, 3049–3067.

Mulhausen, J., Damiano, J., and Pullen, E. L. (2006). "Defining and Judging Exposure Profiles." In *A Strategy for Managing Occupational Exposures*, eds. J. S. Ignacio and W. H. Bullock, 3rd ed. Fairfax, VA: AIHA Press.

Ramachandran, G. (2005). *Occupational exposure assessment for air contaminants*. 1st ed. Boca Raton, FL: CRC Press.
